# Relevance of force-velocity and change of direction assessments for the ranking position in elite junior tennis players

**DOI:** 10.3389/fspor.2023.1140320

**Published:** 2023-02-27

**Authors:** Nicola Reiner Volk, Jo-Lâm Vuong, Alexander Ferrauti

**Affiliations:** Department of Training and Exercise Science, Faculty of Sport Science, Ruhr University Bochum, Bochum, Germany

**Keywords:** sprint, maturation, peak height velocity, acceleration, deceleration, female

## Abstract

**Purpose:**

This study aimed to correlate sprint mechanical parameters (SMP) of a linear sprint (LS) and a tennis specific modified 505 (Tm505) change of direction (CoD) test obtained with a motorized resistance device (MRD) to the current tennis ranking position (RP).

**Methods:**

107 male and 86 female elite junior tennis players nationally ranked in the German Tennis Federation between 10 and 18 years participated in the study. According to their age at peak height velocity (PHV), players were divided into pre-PHV, circa-PHV, and post-PHV groups. SMP were derived from instantaneous time-velocity data of two 20 m all-out LS measured with 333 Hz. Further, mean values from two Tm505 trials with constant 3 kg loading over acceleration-deceleration (1a) and reacceleration (1b) phases were measured with an MRD. SMP of LS and CoD measurements were partially correlated with the current RP in the overall national ranking by controlling for biological maturation.

**Results:**

Low to moderate correlations (*r_s_* = −0.1 to −0.3) were found between SMP and the RP in all male and female age groups. Correlations of the CoD measurements were overall more pronounced, particularly in girls (*r_s_* = −0.44). All linear SMP, like maximal theoretical force (F_0_; N/kg), and maximal theoretical velocity (v_0_; m/s), maximal power (P_max_; W/kg), improved over maturation for both genders with P_max_ being most important for sprint performance. Further, P_max_ was shown to correlate with the girls ranking position (*r_s_* = −0.31). During the Tm505, matured players achieved significantly faster overall total and CoD times. Positioning of CoM before CoD enlarged over maturation and was found to correlate to the RP in both sexes. In addition, nearly all SMP significantly correlated to the primary performance outcomes in the Tm505 test in both genders (*r* = −0.3 to −0.6).

**Conclusion:**

CoD performance has a moderate and higher impact on tennis performance compared to LS. CoD performance as well as P_max_ achieved a higher relevance for the ranking position predominantly in girls compared to boys. Hence, particularly P_max_ as well as the transfer to on-court CoD motor skills should be a central training goal in elite junior tennis players besides technical skills and should depend on maturation status and gender.

## 1. Introduction

Movement speed is considered essential for success in many team and racket sports (1, 2). Movement speed in racket sports consists of several factors, including the capacity to accelerate fast and effectively in various directions. Therefore, precise footwork patterns must be used during the stroke preparation, and quick change of direction must be made (e.g., deceleration, reorientation, and acceleration). Many of these actions happen according to external stimuli that must be detected and processed rapidly (3). Match play analysis at the highest level showed that average running distance ranges between 2.5 and 4.5 m and four changes of direction per point (4), with roughly 20% of these movements involving a medium or high time pressure (5). Players usually cover their backhand side more and therefore sprint frequently towards an open forehand side with a subsequent change of direction after the stroke (5). In addition, more than 70% of movements during tennis match play are lateral, with up to 1,000 direction changes during competitive games (6).

Numerous studies highlight the significance of different speed characteristics as crucial parts of the physical demands of tennis (1, 7, 8). Consequently, various fitness testing for elite tennis players is mandatory, focusing on linear sprint (LS) and change of direction (CoD) tests. These are often part of an extensive testing battery (9). During LS, players need to effectively apply forces to the ground in a way to accelerate forwards (10). Thereby, the highest horizontally orientated forces can be found at the beginning of the sprint. Players increase their speed as they keep accelerating towards the finish line, trying to effectively apply force at high velocities to reach their maximum speed. A 20 m distance is recommended for testing tennis players (11), although running speed could theoretically increase as some players are likely to not reach top speed for the given distance. As a result, an extrapolated linear force-velocity (F-v) and parabolic power-velocity (P-v) relationship may accurately describe the overall mechanical capability to produce horizontal force and velocity at low and high speeds during sprint running (10). Research has shown that sprint performance (e.g., 20 m time) highly depends on the maximal horizontal power output during sprint acceleration (12). On the other hand, change of direction tests are considered independent motor skills with a high degree of neuromuscular and biomechanical specificity (13). Altering the distance and velocity in the initial run-up implies different loading for the player (14). Using reliable tests (e.g., 505) can provide differentiated information for tennis performance since deceleration and acceleration depend on different and numerous muscle actions (15). During the 505 test, athletes accelerate towards a predefined turning point before decelerating to perform a 180° change of direction and sprinting back to the starting line. Additionally, technical abilities should be considered because an efficient deceleration technique is essential to apply optimal braking forces (15).

Regarding movement speed, path analysis has highlighted change of direction speed as the most important factor for tennis performance (ranking position) in elite junior tennis (8). Although linear speed has a minor influence on tennis performance, it was shown to be an essential determinant of CoD speed (8). Unfortunately, most studies have only used light gate measurements for linear sprint and change of direction tests in tennis testing. Testing with light gates requires the athlete to start 0.3 m up to 1 m behind the starting line. Hence, the detection of the trigger signal to start the time measurement represents a flying start which in turn does not reflect the initial force production capabilities of the athlete. Furthermore, the development of movement speed as a consequence of forces acting on the ground, and maximal sprint speed, cannot be detected accurately with only a few split times over a certain running distance. Force and power output are usually overestimated, while maximal speed is underestimated when using split times to calculate SMP. To account for these discrepancies, literature suggests manually adding 0.5 s to the sprint times for all players (16). Similarly, for CoD tests, split times from light gates are the main parameters reported limiting the understanding of deceleration qualities and split times achieved. Due to this methodological limitation, a detailed individual analysis beyond split times was not yet presented in literature. As a result, there is still a lack of information about how tennis players move during linear sprint and CoD tasks as they mature. Using only split times impairs information about how two players might achieve the same respective split times during a LS or CoD. The subsequent training intervention is likely only beneficial for one player. Hence it is necessary to investigate individual strengths and weaknesses of the sprint mechanical parameter and the cause of CoD performance in detail during the maturation process of junior tennis players. Quantifying the relevance of these measurements for tennis performance and to which extent LS abilities might transfer to specific parts of the CoD can provide detailed and individualized information to understand and improve the players physical abilities and, subsequently, tennis performance.

Therefore, the study aimed to detect the influence of sprint mechanical parameters and change of direction performance measurements to the tennis performance indicated by the ranking position for different gender and maturation groups. It was hypothesized that CoD measurements are more prominent and higher correlated to the gender-specific ranking position than SMP and that SMP are correlated to COD performance. Further, the relevance of SMP and CoD parameters differs depending on maturation and gender.

## 2. Methods

### 2.1. Subjects

107 male and 86 female elite junior tennis players nationally ranked in the German Tennis Federation between 10 and 18 years participated in the study. Boys and girls were divided according to their age at peak height velocity (PHV) into pre-PHV (boys: *n* = 42, 12.2 ± 0.8 years, 152.3 ± 6.8 cm, 40.9 ± 5.2 kg), circa-PHV (boys: *n* = 33, 14.1 ± 0.6 years, 170.8 ± 7.4 cm, 56.1 ± 7.8 kg; girls: *n* = 37, 12.3 ± 0.8 years, 157.4 ± 6.8 cm, 45.0 ± 6.7 kg), and post-PHV (boys: *n* = 32, 16.2 ± 0.8 years, 181.9 ± 7.1 cm, 71.3 ± 9.9 kg; girls: *n* = 48, 14.4 ± 1.2 years, 168.3 ± 5.0 cm, 57.2 ± 5.7 kg) groups. The female pre-PHV group consisted of only one player and was therefore not mentioned. Data were collected in the spring of 2022 at the respective national and regional training facilities all over Germany under standardized conditions (indoor hard court) during the regular biannual physical testing of the German national tennis federation (9).

All tested players were selected by the regional coaches as the currently best ones which regularly participated in tennis matches in their age groups and were free from injury on the test day. The players signed a written consent form to participate in the physical test battery and provided their data for *post-hoc* anonymous group statistics. All procedures were in accordance with the declaration of Helsinki. Ethical clearance was provided by the ethics committee of the Ruhr University Bochum, submitted (26.02.2013, No. 4621-13).

### 2.2. Procedure

A standardized test battery consisting of anthropometric and physical performance tests was implemented in 2010 by the National Tennis Federation (The German Physical Condition Tennis Test). Since then, nationally ranked junior players have been recruited twice a year to participate (9). The tests always took place under standardized conditions on indoor courts (hard court surface) in a predefined testing order. After a 15-min individual warm-up, the athletes went through four test stations. All tests were performed in the same sequence and with the same test equipment (9). Within the framework of the standardized test battery linear sprint and change of direction measurements were conducted at the beginning of the testing day (station one). Anthropometric data were taken later on the same day during the test procedure.

### 2.3. Measurements

#### 2.3.1. Anthropometrics

Anthropometric measurements included body weight, body height, and sitting height. Body weight was measured with a digital scale (±0.1 kg, ADE Electronic Column Scales, Hamburg, Germany) and body height with a fixed stadiometer (±0.1 cm, Holtain Ltd., Crosswell, UK). To measure the sitting height, a table was used in addition to the stadiometer. Trained test supervisors performed all measurements in accordance with the ISAK guidelines (17). The timing of puberty was estimated using the maturity offset method (18). Age at the time of maximum linear growth (PHV) indicates somatic maturity (19). Maturity (in years) was obtained by subtracting the chronological age at the time of measurement from PHV. The resulting value (YAPHV) indicates how far the current maturity level is away from the player's PHV. Biological age groups were defined as: pre-PHV (>−1.0 YAPHV), circa-PHV (−1.0 to 1.0 YAPHV), and post-PHV (>1.0 YAPHV).

#### 2.3.2. Ranking position

Overall tennis performance was evaluated in terms of the gender-specific ranking position (RP) on the overall annually published national ranking lists. The rankings were published at the time of the diagnostics. Only players who were tested and positively identified on the national ranking lists were included in the study. The players’ original rankings on the overall list were revised so that the top-ranked player was placed first and the lowest-ranked player last. Male players tested were within the top 1%–94% of the gender-specific rankings, while female players tested were within the top 2%–87%.

#### 2.3.3. Twenty-meter linear sprint and force-velocity profile

##### 2.3.3.1. Sprint testing

Athletes performed two 20 m all-out linear sprints (LS) from a staggered standing stance with 2–3 min of passive recovery between trials to ensure no fatigue-related performance decrease (9). All sprints were commenced from a static position, meaning that leaning backward before accelerating forward was not allowed. After the test leader gave a ready signal, the athletes started on their own initiative. Instantaneous velocity-time data were recorded (333 Hz) using a motorized resistance device (MRD; 1080 Sprint, 1080 Motion, Lidingö, Sweden). The MRD was placed 3 m behind the starting line, with the cord attached to the athlete by a centrally located ring (sacrum) on a belt firmly tightened around the pelvis. A resistance of 1 kg was applied over the entire sprint. The no-flying weight mode was selected because the 1080 Sprint offers different modes. Settings were controlled by a computer application (1080 Sprint, 1080 Motion, Lidingö, Sweden). The average values of the two trials for 5, 10, 20 m split times and the average speed of the best 5 m during the sprint (peak velocity) were taken as performance measurements.

##### 2.3.3.2. Force-velocity profile

Each sprint was separately analyzed using the velocity-time raw data to compute sprint mechanical parameters (SMP). The method used was previously described in detail (10, 20, 21). Briefly, this computation method using the raw data is based on a macroscopic inverse dynamics analysis of the center-of-mass motion. Instantaneous velocity data were combined with the system mass and aerodynamic friction to compute the athlete's propulsion capacities over different velocities. These can be described by the individual linear horizontal F-v-Profile. F-v-Profiles were extrapolated to calculate relative theoretical force (F_0_: N/kg, force capabilities), theoretical maximal velocity (v_0_: m/s, velocity capabilities), and maximal power output (P_max_: W/kg, power capabilities) in the antero-posterior direction. The ratio between the independent variables F_0_ and v_0_ corresponds to the slope (S_Fv_) of the F-v-relationship. Further, “technical” abilities can be computed as the maximal ratio of the horizontal force applied to the ground (RF_max_) and its rate of decrease as velocity increases (D_RF_) (21). The average values of the two trials were taken for further analysis. Due to injuries or no valid ranking position on the test day, three male and four female players were not able to perform the LS and were subsequently excluded from the analysis.

#### 2.3.4. Tennis modified 5-0-5 change of direction test

**Figure 1:**
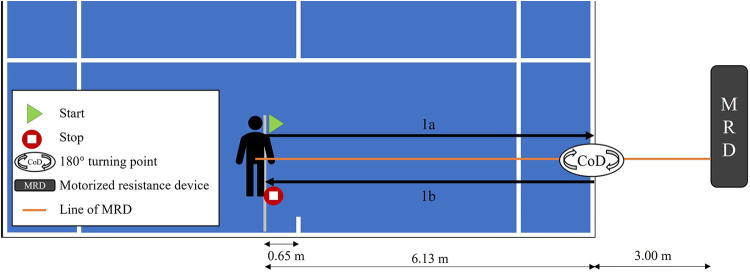
Set-up of the modified 505 change of direction test (Tm505) on a tennis court.

In addition, a modified version of the 505 change of direction test according to the demands of the tennis court was introduced (Tm505; Figure 1). Athletes performed two all-out trials only to their forehand side. Instantaneous velocity-time data were recorded using a MRD (333 Hz; 1080 Sprint, 1080 Motion, Lidingö, Sweden). Participants started in a ready position facing the net on the ad-court side of the indoor hard-court (right-handed) with both their feet planted on the floor. The starting line was positioned 0.65 m laterally from the center service line. The MRD was placed 3 m perpendicular behind the doubles sideline to allow the Tm505 to be performed on the tennis court. The string from the MRD was attached to the athlete's pelvis using a pear-shaped carabiner and a tightly laced sling rope. The tightening knot was placed on the contralateral side of the turning foot to allow an undisturbed swivel of the carabiner. A permanent loading of 3 kg was applied to the participant using the built-in servo motor (2000 RPM OMRON G5 Series Motor; OMRON Corp., Kyoto, Japan) according to the reliability analysis of Eriksrud and colleagues (22). The no-flying weight mode was selected because different modes are offered by the 1080 Sprint. Settings were controlled by a computer application (1080 Sprint, 1080 Motion, Lidingö, Sweden). Due to injuries, no valid ranking position on the test day, or incorrect execution, eleven male and ten female players were not able to perform the Tm505 and were subsequently excluded from the analysis.

After the ready signal from the test leader, the athletes started on their own initiative. Any movement of the center of mass (CoM) greater than 0.2 m/s initiated the time measurement. From this, the entry phase before the CoD was defined as the acceleration and deceleration towards the doubles sideline (1a). From the moment of the CoD, the reacceleration phase started until the end of the test (1b). A successful trial was confirmed when the athlete at least touched the doubles sideline with their outside foot and ran across the starting line. The following performance outcome measurements were obtained during the Tm505 test with respect to Eriksrud and colleagues (22): total time (Tm505_time_), total distance (Tm505_dist_), time phase 1a (Tm505_1a_time_), average velocity during phase 1a (Tm505_1a_vel_), time phase 1b (Tm505_1b_time_), average velocity during phase 1b (Tm505_1b_vel_), the distance of the CoM to the CoD point at 0.5 s before the CoD (Tm505_dist_−0.5s_), the time for a fixed distance (1.37 m; distance between singles and doubles sideline) before and after the CoD (; Tm505_time_CoD_), and the time needed from peak velocity during phase 1a to stop before CoD (Tm505_time_decel_).

### 2.4. Statistical analysis

Raw data sets of each trial for LS and CoD were individually processed using custom-made R scripts (Rstudio, PBC, Boston, MA; version 4.1.3) and Microsoft Excel (Microsoft Corp., Redmond, WA, United States) spreadsheets. Mean values of trials for the variables of interest were used for further analysis. The overall gender-specific ranking position was taken from the annually published ranking lists of the German Tennis Federation. The highest-ranked player in the overall gender-specific ranking who participated was assigned first place and the lowest-ranked player last. Main statistics were done using JAMOVI (The jamovi project, Sydney, Australia; version 2.2.5.0). Spearmans’ Rho rank correlation (*r_s_*) was used to correlate the performance measurements of linear sprint and change of direction with the external criterion of ranking position. Biological maturation (YAPHV) was partialized out to account for maturation status. Further, linear sprint and change of direction measurements were partially correlated using Pearson's correlation (*r*) analysis by controlling for biological maturation (YAPHV). The following magnitude thresholds were applied: <0.10, trivial; from 0.10 to 0.30, small; from 0.30 to 0.50, moderate; from 0.50 to 0.70, large; from 0.70 to 0.90, very large; and from 0.90 to 1.00, almost perfect (23). A significance level of *α *= 0.05 was accepted.

## 3. Results

**Figure 2:**
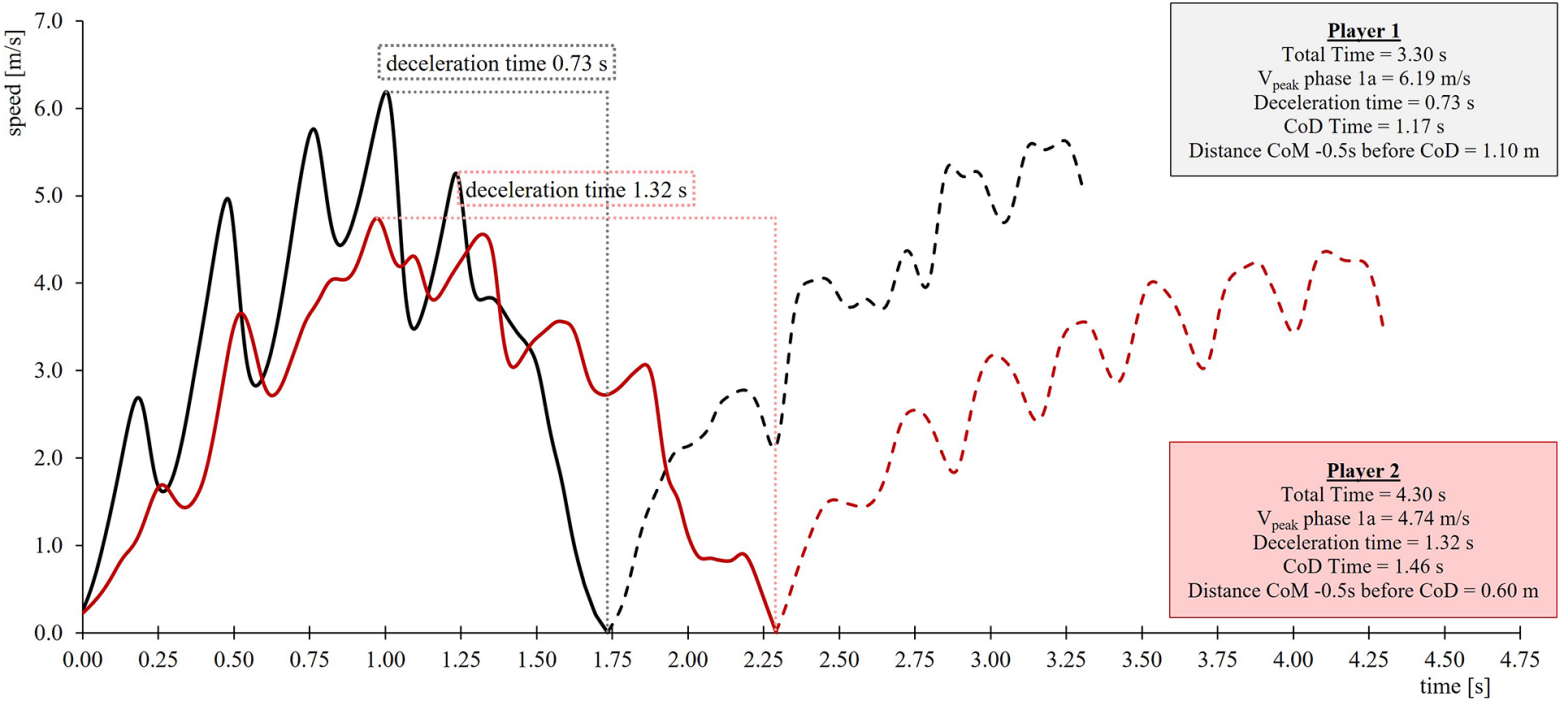
Velocity-time data from the Tm505 for two players with different total times, peak velocities, deceleration and change of direction times, and distances of their CoM at a fixed time point (0.5s) prior to the CoD. Player 1 represents a male player from the post-PHV, and Player 2 is from the male pre-PHV group. Solid lines correspond to phase 1a, dashed lines phase 1b, dotted lines visualize the deceleration time from vpeak to the point of CoD.

The main results are presented as mean ± standard deviation in Table 1. Matured male and female athletes represent faster split times over all distances. All SMP improve from pre-PHV to post-PHV for both genders. Changes between maturation groups are generally more pronounced in males than females. Especially, P_max_ is emphasized more than other SMP over maturation. Changes in v_0_ mainly increase compared to F_0_. Consequently, the slope of the F-v-Profile (S_Fv_) becomes less negative over maturity (Figure 2). Regarding the specific tennis-modified change of direction task, matured boys and girls achieve faster total times along with better CoD times (Table 1). Phase-specific results show more substantial improvement in phase 1b compared to phase 1a. The distance of the CoM 0.5s prior to the CoD increased by 12% for females and by 23% for males over maturation.

**Table 1:** Descriptives presented as mean ± standard deviation of linear sprint performance and mechanical as well as change of direction parameters split by sex and biological maturation.

	Male			Female	
	pre-PHV	circa-PHV	post-PHV	circa-PHV	post-PHV
**Athlete characteristics**					
Age (years)	12.2 ± 0.8	14.1 ± 0.6	16.2 ± 0.8	12.3 ± 0.8	14.4 ± 1.2
Mass (kg)	40.9 ± 5.2	56.1 ± 7.8	71.2 ± 9.9	45.0 ± 6.7	57.2 ± 5.7
Height (cm)	152.3 ± 6.8	170.8 ± 7.4	181.9 ± 7.1	157.4 ± 6.8	168.3 ± 5.0
**Sprint times and peak velocity**					
5 m (s)	1.50 ± 0.11	1.38 ± 0.07	1.31 ± 0.07	1.45 ± 0.08	1.41 ± 0.09
10 m (s)	2.45 ± 0.15	2.24 ± 0.10	2.11 ± 0.10	2.38 ± 0.11	2.29 ± 0.12
20 m (s)	4.16 ± 0.25	3.77 ± 0.16	3.51 ± 0.15	4.04 ± 0.19	3.84 ± 0.19
peak velocity (m/s)	5.99 ± 0.41	6.58 ± 0.50	7.27 ± 0.66	6.06 ± 0.54	6.47 ± 0.55
**Force-velocity-characteristics**					
F_0_ (N/kg)	7.53 ± 1.02	8.10 ± 0.80	8.50 ± 0.79	7.69 ± 0.78	7.80 ± 0.75
v_0_ (m/s)	6.34 ± 0.49	7.03 ± 0.42	7.73 ± 0.48	6.48 ± 0.44	6.90 ± 0.43
P_max_ (W/kg)	12.0 ± 2.46	14.2 ± 1.67	16.4 ± 1.94	12.5 ± 1.54	13.5 ± 1.75
S_Fv_ (−F_0_/v_0_)	−1.19 ± 0.12	−1.16 ± 0.14	−1.10 ± 0.12	−1.19 ± 0.14	−1.13 ± 0.11
RF_max_ (%)	57.4 ± 4.64	60.5 ± 3.44	62.4 ± 3.31	58.4 ± 3.76	59.1 ± 3.42
D_RF_ (%)	−9.0 ± 0.79	−8.2 ± 0.95	−7.6 ± 0.77	−8.9 ± 0.98	−8.3 ± 0.73
**Change of direction characteristics**					
Tm505_time_ (s)	4.12 ± 0.24	3.78 ± 0.15	3.58 ± 0.11	3.99 ± 0.20	3.82 ± 0.17
Tm505_dist_ (m)	12.37 ± 0.37	12.23 ± 0.29	12.02 ± 0.24	12.40 ± 0.41	12.15 ± 0.22
Tm505_1a_time_ (s)	2.14 ± 0.14	2.01 ± 0.10	1.95 ± 0.09	2.10 ± 0.12	2.04 ± 0.10
Tm505_1a_vel_ (m/s)	2.91 ± 0.19	3.05 ± 0.15	3.09 ± 0.15	2.96 ± 0.14	2.98 ± 0.14
Tm505_1b_time_ (s)	1.98 ± 0.14	1.77 ± 0.09	1.63 ± 0.09	1.89 ± 0.11	1.78 ± 0.10
Tm505_1b_vel_ (m/s)	3.15 ± 0.21	3.46 ± 0.17	3.71 ± 0.17	3.29 ± 0.14	3.43 ± 0.20
Tm505_dist −0.5s_ (m)	0.81 ± 0.21	0.93 ± 0.14	0.98 ± 0.14	0.81 ± 0.16	0.91 ± 0.13
Tm505_time_CoD_ (s)	1.38 ± 0.10	1.28 ± 0.06	1.21 ± 0.04	1.34 ± 0.05	1.29 ± 0.06
Tm505_time_decel_ (s)	1.04 ± 0.12	0.98 ± 0.10	0.92 ± 0.10	1.03 ± 0.10	0.99 ± 0.08

Peak velocity (m/s), averaged velocity over best 5 m; relative theoretical force, F_0_; theoretical maximal velocity, v_0_; maximal power output, P_max_; slope of F-v-Profile, S_Fv_; maximal ratio of the horizontal force applied to the ground, RF_max_; decrease in RF, D_RF_; total time, Tm505_time_; total distance, Tm505_dist_; time phase 1a, Tm505_1a_time_; average velocity phase 1a, Tm505_1a_vel_; time phase 1b, Tm505_1b_time_; average velocity phase 1b, Tm505_1b_vel_; distance of CoM 0.5 s before the CoD, Tm505_dist_−0.5s_; time for a fixed distance of 1.37 m before and after the CoD, Tm505_time_CoD_; time from peak velocity in phase 1a to CoD, Tm505_time_decel_.

Low to moderate significant correlations (*r_s_* = −0.22 to −0.33) were found between SMP and RP across all male and female athletes (Table 2). However, separated by gender, low significant correlations between SMP and RP were found in females, whereas none were found in males. Regarding sprinting performance (20 m time), P_max_ showed the highest correlation of all SMP in both males (*r* = −0.77) and females (*r* = −0.86). During the sprint acceleration, the correlations of F_0_ and v_0_ to the split times vary in opposite directions as a function of sprint length. The correlations of F_0_ decrease while values for v_0_ increase for longer sprint distances. Unlike linear sprint measurements, significant correlations of CoD parameters to the RP were generally more pronounced (*r_s_*_ _= −0.24 to 0.44). Correlations of CoD measurements to RP are more dominant in females than males (Table 3). The total time (Tm505_time_) and the time of the exclusive change direction time (Tm505_time_CoD_) represent the highest correlations to RP (*r_s_*_ _= 0.44) in girls. With respect to the Tm505_time_CoD_, the distance of the CoM prior to the CoD displays low to moderate significant correlations to the RP (*r_s_*_ _= −0.24 to −0.31) for both sexes. In addition, F_0_, v_0_, P_max_, and RF_max_ significantly correlate to the main performance outcomes in the Tm505 test (Tm505_time_ and Tm505_time_Cod_) in both genders (*r* = −0.3 to −0.6).

**Table 2:** Partial correlation of mechanical sprint parameters to gender specific ranking position split by sex and in combination while controlled for years away from peak height velocity (YAPHV).

	Male (*n* = 104)		Female (*n* = 82)	
	*r_s_*	sig	*r_s_*	sig
F_0_ (N/kg)	−0.13		−0.20	
v_0_ (m/s)	−0.05		−0.33	**
P_max_ (W/kg)	−0.11		−0.31	**
S_Fv_ (−F_0_/v_0_)	0.15		0.05	
RF_max_ (%)	−0.13		−0.22	*
D_RF_ (%)	0.06		−0.01	

Controlling for YAPHV.

* *p* < 0.05.

** *p* < 0.01.

*** *p* < 0.001.

**Table 3:** Partial correlation of change of direction performance parameters to gender specific ranking position split by sex and in combination while controlled for years away from peak height velocity (YAPHV).

	Male (*n* = 96)		Female (*n* = 76)	
	*r_s_*	sig	*r_s_*	sig
Tm505_time_ (s)	0.10		0.44	***
Tm505_dist_ (m)	−0.09		0.08	
Tm505_1a_time_ (s)	0.09		0.31	**
Tm505_1a_avgvel_ (m/s)	−0.11		−0.35	**
Tm505_1b_time_ (s)	0.10		0.27	*
Tm505_1b_avgvel_ (m/s)	−0.13		−0.26	*
Tm505_dist_−0.5s_ (m)	−0.24	*	−0.31	**
Tm505_time_CoD_ (s)	0.25	*	0.44	***
Tm505_time_decel_ (s)	0.01		0.27	*

Controlling for YAPHV.

* *p* < 0.05.

** *p* < 0.01.

*** *p* < 0.001.

## 4. Discussion

This study aimed to correlate sprint mechanical parameters of a linear sprint (LS) and a tennis-specific change of direction test (Tm505) obtained with a motorized resistance device to the current gender-specific ranking position and the maximum sprinting performance of elite junior male and female junior tennis players. The main finding of this study is that correlations of the Tm505 to the gender-specific ranking position are generally more pronounced than LS parameters in youth tennis players. SMP like F_0_, v_0_, P_max_, and RF_max_ significantly correlate to the main performance outcomes in the Tm505 in both genders. P_max_ was shown to correlate significantly with the girls ranking position as well.

To the best knowledge of the authors, this paper is the first to look at the correlation of sprint mechanical parameters to the RP in elite youth tennis players. Previous literature has attempted to identify crucial physical performance contributors to the ranking position of youth tennis players (8, 24). However, different split times were mainly used during a 20 m all-out sprint test or change of direction test which limit the informative value about how the players move. This lack of information can be covered by using a more detailed measure and analysis of the sprint. Using a valid MRD (25) combined with Samozino and colleagues (10) simplification model allowed us for the first time to evaluate the sprint mechanical properties of the sprint acceleration of different age groups and both sexes in junior tennis. Low to moderate correlations of F-v-parameters to the RP were found for players (Table 2). According to the results of Samozino and his research group, sprint acceleration performance is directly related to the average power output in the horizontal direction throughout the sprint and has the most impact on sprint performance (12). Our results are consistent with this research showing the highest impact of P_max_ on sprint performance. In the present study, superior values of P_max_ correlate to a better ranking position, particularly in girls (*r_s_*_ _= 0.31).

The present findings indicate that the overall importance of linear speed for the national ranking is relatively low. These findings are in accordance with previously reported results (8). This can mainly be attributed to the dimensions of the tennis court and the typical short distances for acceleration and deceleration. Additionally, the high demands of technical and tactical skills are most likely dominating over the athletic abilities in junior tennis. In this regard, previous studies have shown that sport specific-skills, e.g., serve speed, showed the highest correlations to ranking position (24). These consistent results suggest that technical demands are paramount in tennis players compared to linear sprint measurements. However, the results of the Tm505 highlight a stronger relevance as performance limiting factors compared to the linear sprint performance (Tables 2, 3). Especially in girls, the Tm505_time_ and Tm505_time_CoD_ demonstrate the highest significant correlations to the RP (*r*_*s*_* *= 0.40 and *r_s_*_ _= 0.44, respectively; Table 3). These results are in agreement with current studies reporting CoD qualities as the most relevant factor for tennis performance (8, 7). During tennis matches, players must change their direction by running primarily toward the forehand side. They may also run into a backhand or a ball near the net, recovering to the baseline in between. With four CoDs per point and a majority of distances between 2.5 and 4.5 m, 20% of these actions must be completed quickly (4, 26). The specific importance of the CoD performance in girls for reaching a higher-ranking position can be attributed to the different playing styles of men and women during match play and the specificity of the external loads in elite female tennis (4). Since service velocities are far below the values obtained in men's tennis, the game of females usually includes longer rallies with more change of directions, whereas the games of men are more powerful with shorter rallies (4). Additionally, an increasing speed of baseline shots, especially with the two-handed backhand, dominates the female baseline rallies, forcing them to perform under progressive time pressure to move fast during and after changes of direction. Recent research pointed out that elite junior players had better developed speed-accuracy trade-offs than sub-elite players (27). Given these demands, superior CoD abilities along with good speed capabilities might be more advantageous for ranking potion particularly in females.

Linear sprint qualities might influence the ability to execute a good CoD to a certain amount (8). Harper et al. (15) consolidate various strength qualities, potentially contributing to enhanced CoD performance. In this regard, these results confirm improved CoD performance in mature players representing increased speed and strength levels (Table 1). The present findings demonstrate a significant correlation of F_0_, v_0_, P_max_, and RF_max_ to the primary performance outcomes in the Tm505 test without meaningful differences between gender. Interestingly, both P_max_ and v_0_ show the highest correlations amongst SMP to the Tm505_time_ (*r* ≈ −0.56) and Tm505_time_CoD_ (*r* ≈ −0.50) across all tested athletes. Similarly, force-related SMP like F_0_ and RF_max_ are significantly correlated to Tm505_time_ (*r* ≈ −0.51) and Tm505_time_CoD_ (*r* ≈ −0.35). The contribution of different linear sprint abilities might vary over the course of the Tm505. In detail, the Tm505 is divided into the entry phase (1a) and the reacceleration (1b) after the CoD. The former consists of initial acceleration and subsequent deceleration, while the latter is entirely acceleration dependent. Consequently, phases should be considered separate (*r* = 0.28) with respect to technical and physical demands. All the above-mentioned SMP measurements correlate to a higher amount to phase 1b compared to 1a, independent of gender. This seems rational since SMP are measurements of acceleration and not deceleration. Despite initial accelerations in both phases, the amount and orientation of force in the horizontal direction correlates more with phase 1b (*r* ≈ 0.50). This is probably due to better shin angels immediately after the CoD and no deceleration component influencing the time. Better propulsion during linear sprinting can therefore be transferred specifically to a CoD. After the CoD, higher values of P_max_ and v_0_ correlate to a better time for phase 1b in both genders (*r* ≈ 0.57). In essence, P_max_ peaks during full acceleration without deceleration components and is a measure of the overall power output during sprint acceleration (12). The transfer of linear speed mainly to the acceleration phase during CoD was described previously (8). Over the short re-acceleration distance, higher P_max_ values are crucial for a better CoD performance. This is highlighted by the fact that boys and girls improved their time for phase 1b twice as much, which can predominantly be attributed to the improvements in P_max_.

**Figure 3:**
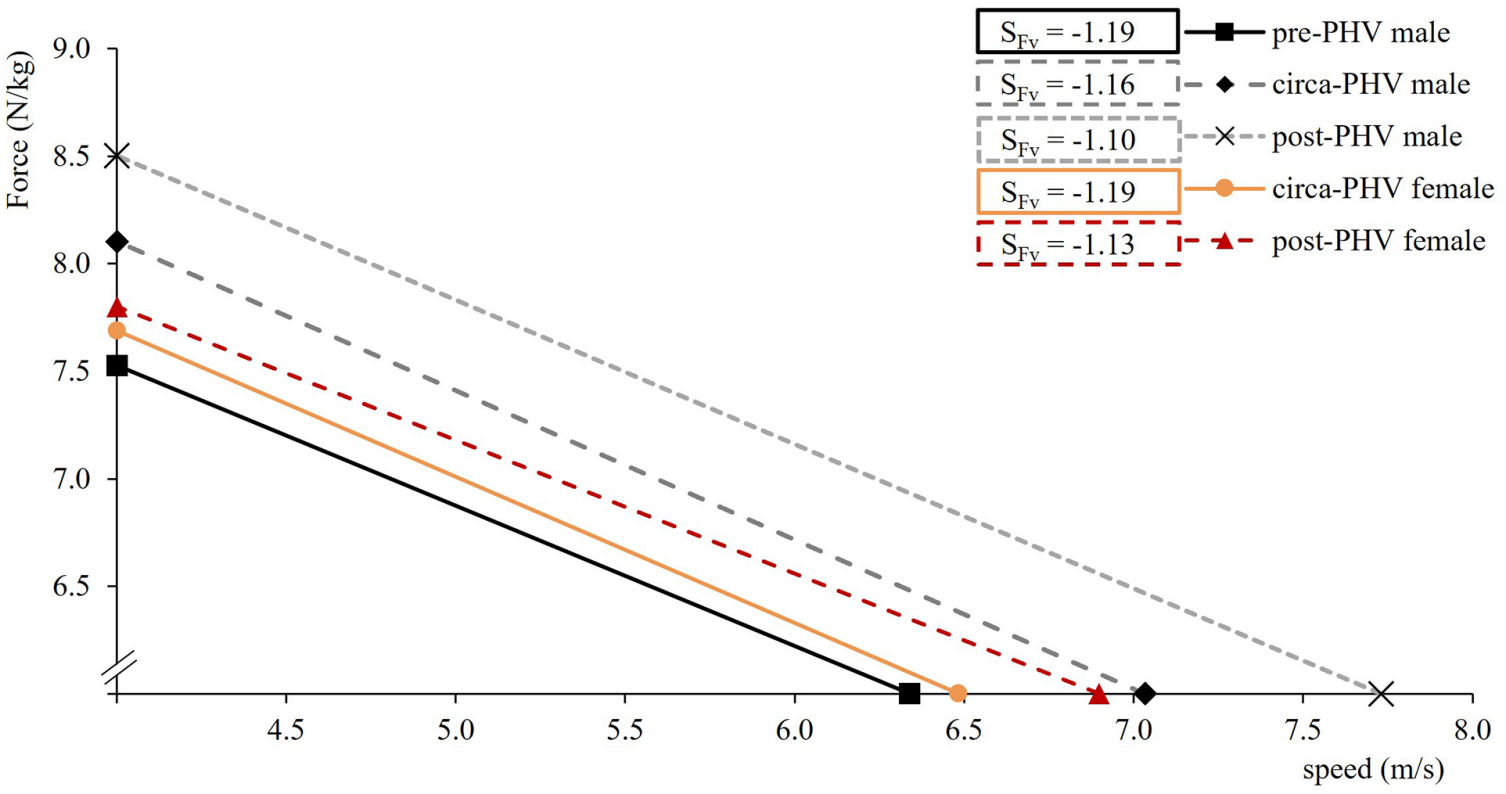
F-v-Profiles for male and female players according to their maturation status. Please note the scales of the *x*- and *y*-axis.

The ability to change direction quickly and efficiently in multiple directions can be considered a determining factor in tennis performance (8). A more prominent open forehand can be observed during match play in recent years, along with change of directions appearing mostly to the forehand side (5). The accompanying higher speeds prior to the CoD require adequate neuromuscular and biomechanical qualities to optimize braking impulse to achieve the desired reduction in whole-body momentum (15). With the intention of testing these demands, athletes were pulled with additional 3 kg loading toward the doubles sideline. Due to the amplified speed, enhanced braking forces paired with improved kinematic positions are essential. A current review suggests, besides other factors, an increased posterior CoM position relative to the lead braking foot as an efficient strategy (15). Consequently, this allows the athlete to apply more horizontally orientated braking forces, thereby prolonging the time in which these forces can be applied (15). In addition, generating more eccentric force is a critical requirement for developing high levels of concentric force in tasks requiring rapid countermovement (e.g., CoD) which might relate to overall better deceleration abilities (15) and is associated with superior CoD performances (14). Improvements can be found in Tm505_1a_time_ and Tm505_1b_time_, and overall time (Table 1). All mentioned time measurements of the Tm505 do correlate in female players (Table 3) indicating a positive influence of elevated acceleration and deceleration capacities for the tennis performance. The relevance of deceleration capacities for the RP in both genders is supported by the significant correlation of the time to decelerate (*r_s_*_ _= 0.25–0.44, Table 3). It is reasonable to assume that matured players optimized their strategy during the CoD tasks, especially during deceleration (Figure 3). The enlarged distance at a fixed time point (0.5s) before the CoD (21%; Table 1) can be interpreted as a measure of CoM position shortly before the CoD, which benefits the task (15). This favorable kinematic position correlates to the RP in boys and girls (Table 3).

**Figure 4:**
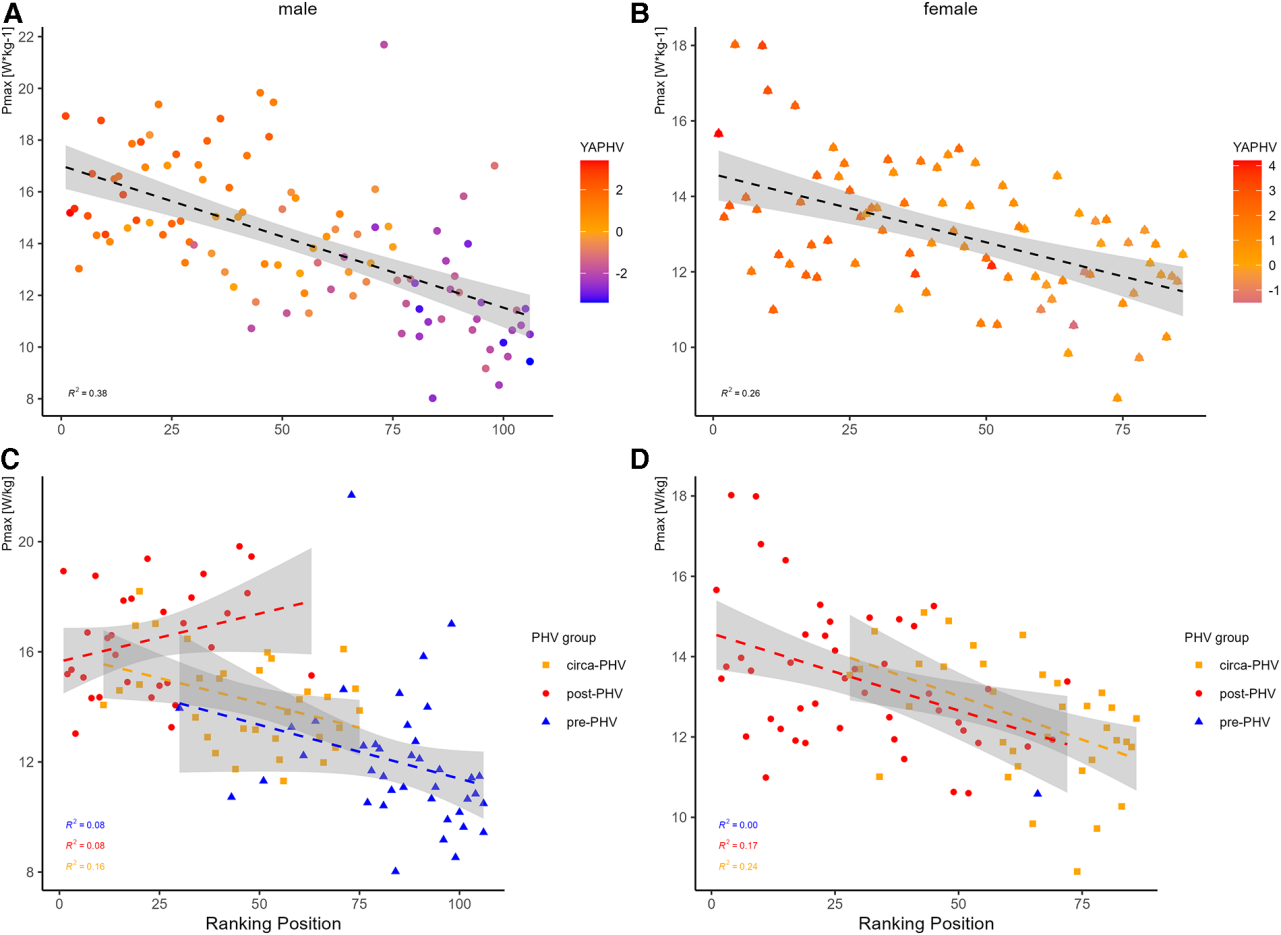
Correlation of p_max_ (W/kg) to gender-specific ranking position taking YAPHV into account (**A,B**) and with reference to PHV groups (**C,D**).

During maturation, boys and girls naturally improve their sprint acceleration and CoD (Table 1). The greatest improvements can be observed in boys from pre-PHV to circa-PHV. Male and female players develop a more velocity-oriented F-v-Profile over maturation (Figure 2), which is mainly responsible for the increase in P_max_. Under the consideration of YAPHV, mature players generally rank better with increased levels of P_max_, and this is statistically significant in girls (Table 3) but can also be seen in boys (Figure 4A,B). However, the post-PHV group in males is most likely responsible for disrupting the overall likewise correlation to the RP (Figures 4C). Until this point, comparable regression lines between similar performance levels (e.g., pre-PHV males and circa-PHV females) can be found. This seems reasonable since women mature earlier than males but have lower physical performance levels due to physiological differences. Thus, the influence of physical development is more evident in boys than girls. In line with previous literature, relative F_0_ and RF_max_ demonstrate only minor changes, while changes in v_0_ are more pronounced (28). Recent research suggests that the increase in body mass (including muscle mass) from circa-PHV to post-PHV in males did not likely have a positive effect on relative maximal strength, leading to no improvement in the ability to apply relative horizontal force at low velocities (28). In the same way, matured players rank better in the Tm505 time measurements (supplements). Albeit only significant correlations were mainly found in girls, similar data distribution was displayed in boys. Again, pots-PHV males seem to develop less in phase 1a than 1b, so total time is influenced. Tm505_time_CoD_ continuously develops across maturation groups in both genders.

The present findings are essential for both talent identification and age- and gender-specific youth training. As stated previously (8), speed and change of direction are multifactorial for tennis performance. Thus, appropriate testing procedures to access specific qualities during linear and change of direction are crucial. Profound diagnostics provide information about strengths and weaknesses in particular parts (e.g., deceleration) and operate as indicators for tailored training prescriptions. The present outcomes are in line with previous results indicating that the improvement of CoD should be prioritized (8). Training of linear speed can reinforce CoD performance and act as subcomponents in this context, especially in the reacceleration phase. Fundamentally, this requires the implementation of appropriate training interventions to improve acceleration and deceleration effectively and thus create the foundation to develop CoD performance. Training should be advised based on the individual needs of the athlete. Practitioners should aim to improve the lower limbs’ force-producing capabilities, technique, and coordination in junior tennis players during maturation to enhance performance (10, 29). Additionally, testing and training motor abilities and skills are essential to consider when discussing performance improvements during maturation. However, challenging to test this, these factors might be a valuable contributor to understanding performance differences. Successful training regimes include methods such as coordination, specific resistance training, maximum strength training, plyometric training, and resisted or assisted sprint training (8, 15, 30, 31). Besides physicality, improvements in deceleration technique might further positively influence performance (15). On this basis, the present results provide indications of a more posterior body position shorty before the CoD linked with a better performance. Regardless of the method used, when implying such interventions, consideration should be given to (training) age and individual maturity status. Better sprint and CoD performance can result in using different strokes and having more choices during the rally because the player has more time to prepare for the stroke. Moreover, tennis performance is a very complex phenomenon, and tennis-specific movements in a game-like context are mandatory to achieve the required performance on the court.

## 5. Conclusion

Conclusively, CoD performance has a moderate and higher impact on tennis performance compared to linear sprint. CoD performance as well as maximal horizontal power (P_max_) achieved, show a higher relevance for the ranking position in girls compared to boys. SMP partly explain the CoD performance, with P_max_ showing the highest correlation to the CoD performance. Besides, all other more pronounced SMP mainly improve the acceleration phase. From a kinematic perspective, a more posterior positioning of the CoM shortly before the CoD positively benefits the deceleration to improve overall CoD performance. During maturation, players primarily improve their P_max_ due to increases in v_0_ alongside all other F-v-metrics resulting in better overall sprint and CoD performance. Hence, the development of maximal power as well as the transfer to on-court CoD motor skills, should be a central training goal in elite junior tennis players. Further, CoD testing should be considered an important marker for performance as well as talent identification, especially in girls. Detailed analysis of the CoD can reveal in-depth insight into how players move and show strengths and weaknesses to prioritize and adjust training programming to improve overall tennis performance. It should be noted that these statements can only be made for the forehand side (open stance) and not the backhand side, where open and closed stances occur. Further, technical and tactical skills should not be neglected since tennis is a prominent technical and tactical sport. A direct transfer from isolated tests might be limited because of the void of cognitive and reactive components.

## Data Availability

The original contributions presented in the study are included in the article/**Supplementary Material**, further inquiries can be directed to the corresponding author.
