# Supplementary figures and images for: Relevance of force-velocity and change of direction assessments for the ranking position in elite junior tennis players
Source: Front Sports Act Living. 2023 Feb 27;5:1140320. doi: 10.3389/fspor.2023.1140320 (PMC10009273; doi:10.3389/fspor.2023.1140320)

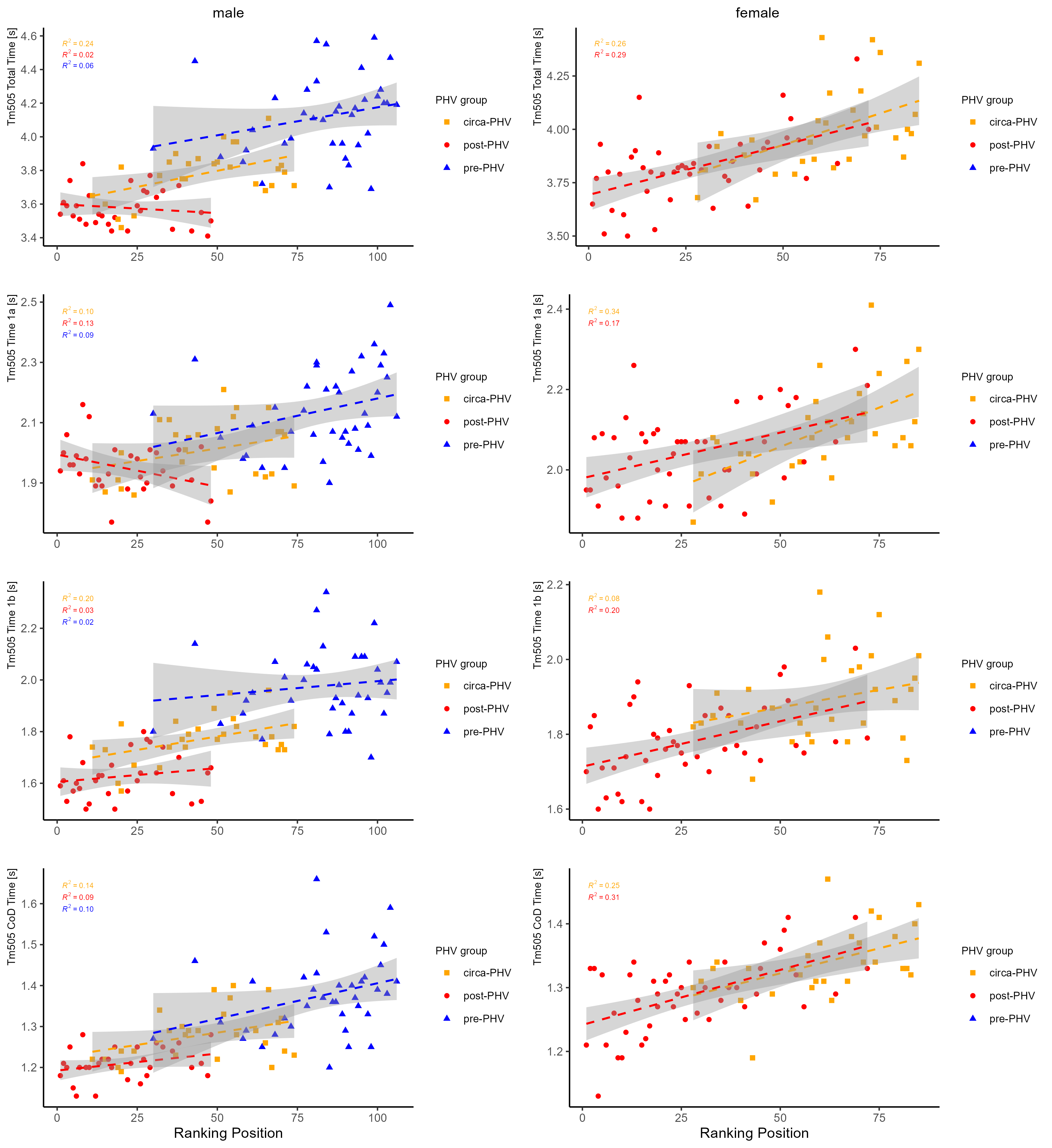

Supplement: Supplementary file 2 [file Image1.jpeg]

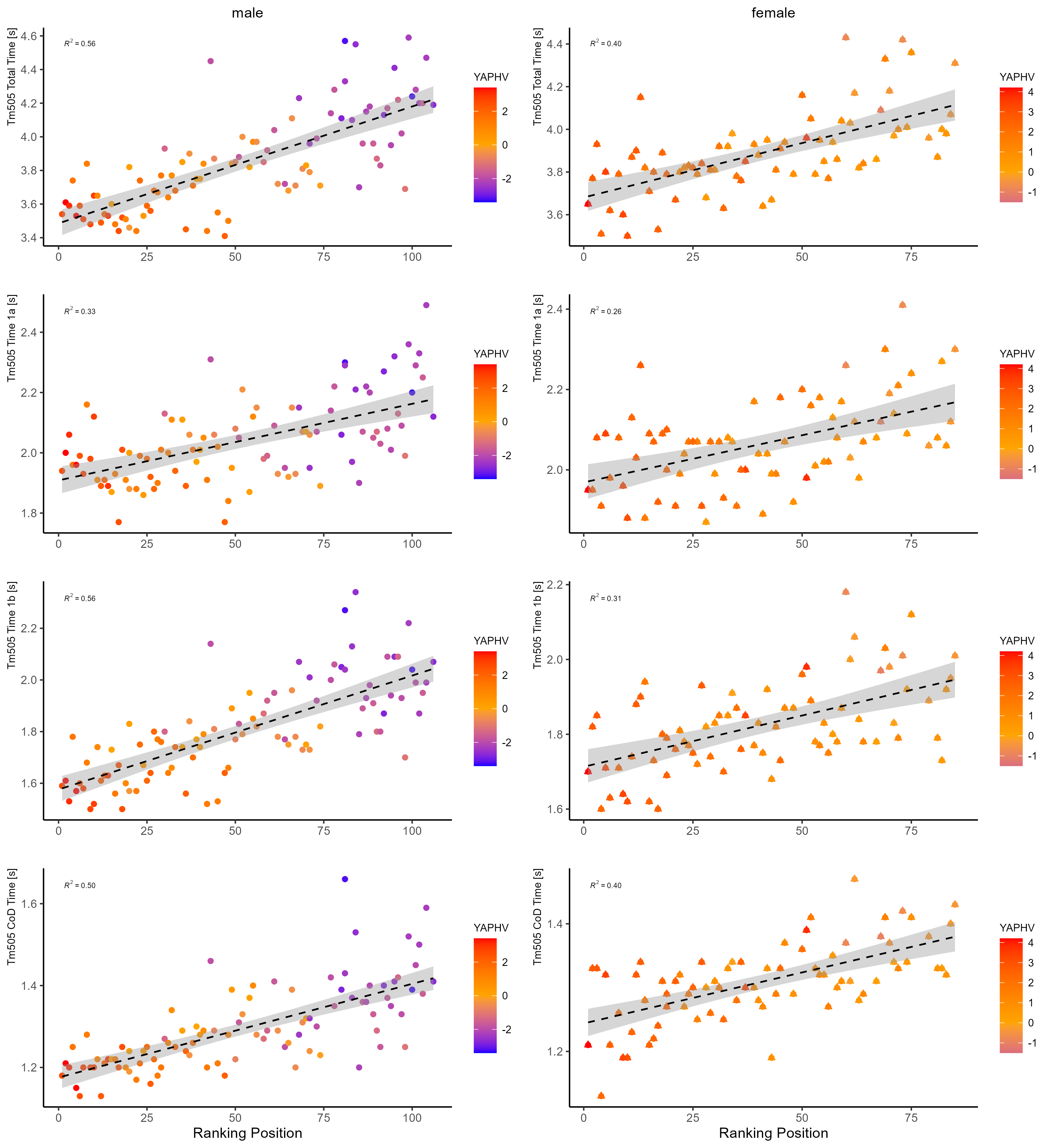

Supplement: Supplementary file 3 [file Image2.jpeg]

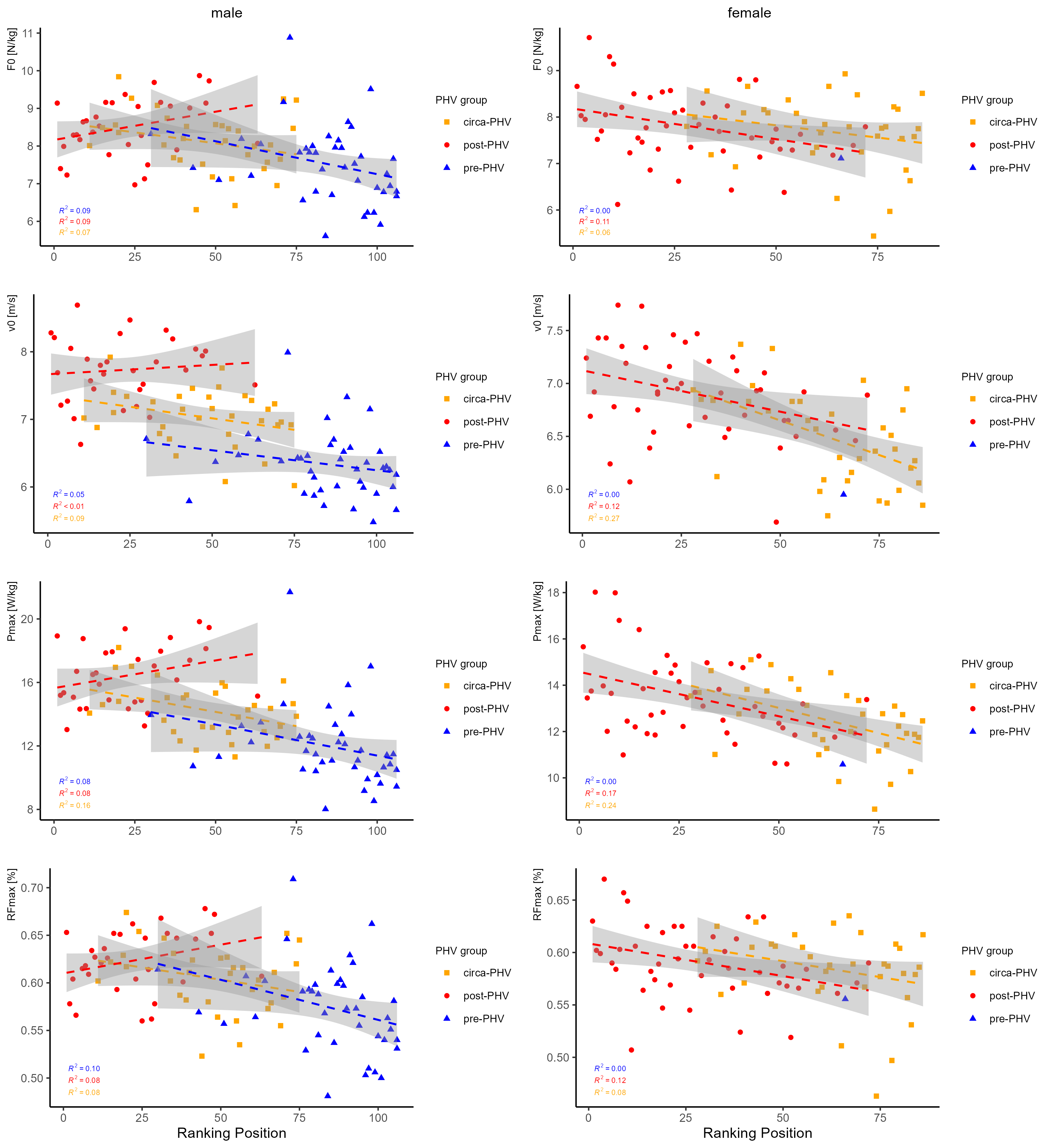

Supplement: Supplementary file 4 [file Image3.jpeg]

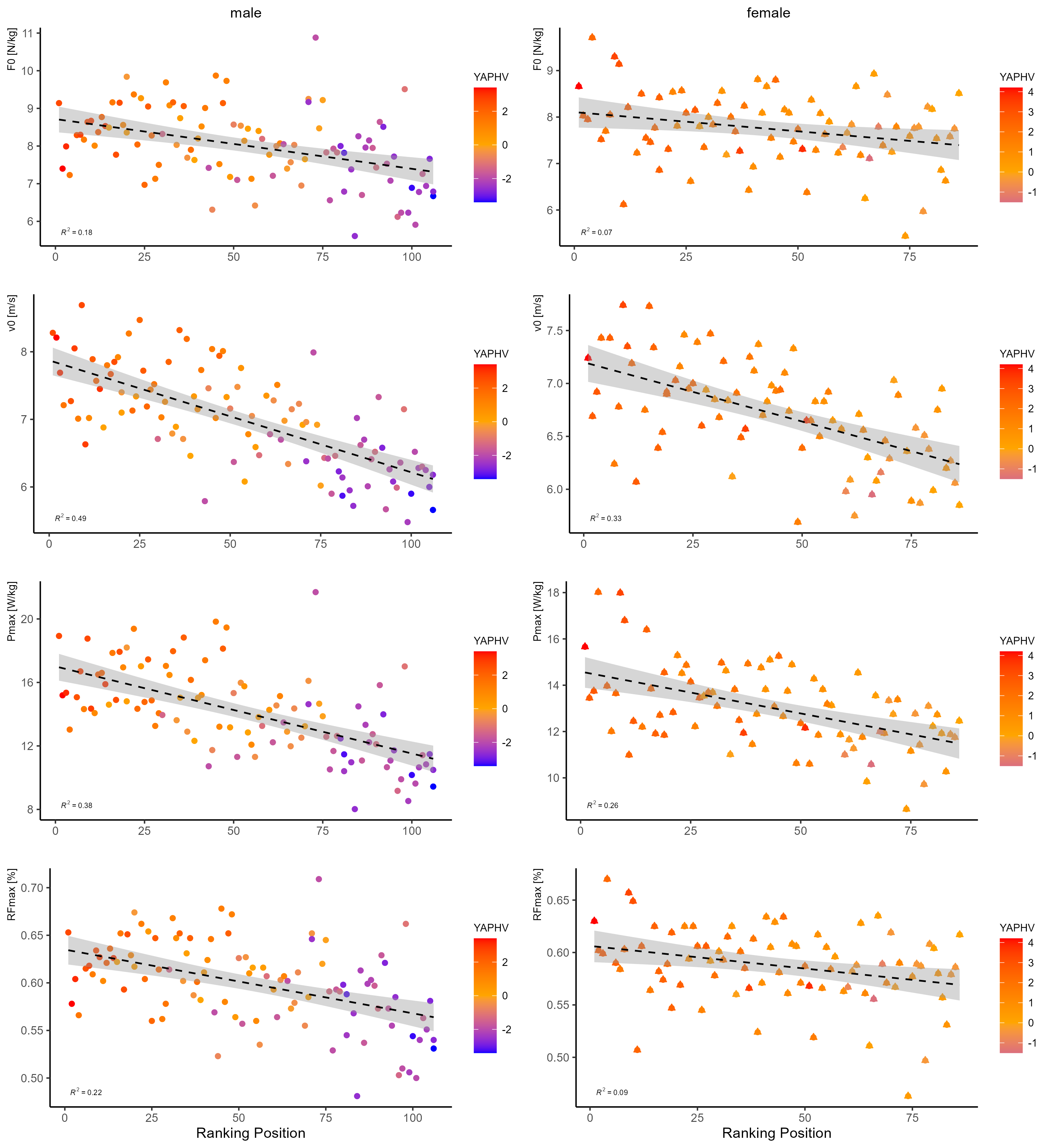

Supplement: Supplementary file 5 [file Image4.jpeg]
